# BPH Procedural Treatment: The Case for Value-Based Pay for Performance

**DOI:** 10.1155/2008/954721

**Published:** 2008-12-21

**Authors:** Mark Stovsky, Irina Jaeger

**Affiliations:** Department of Urology, Case Western Reserve University School of Medicine, University Hospitals Case Medical Center, 11100 Euclid Avenue, Cleveland, OH 44106, USA

## Abstract

The concept of “pay for performance” (P4P) applied to the practice of medicine has become a major foundation in current public and private payer reimbursement strategies for both institutional and individual physician providers. “Pay for performance” programs represent a substantial shift from traditional service-based reimbursement to a system of performance-based provider payment using financial incentives to drive improvements in the quality of care. P4P strategies currently embody rudimentary *structure* and *process* (as opposed to *outcomes*) metrics which set relatively low-performance thresholds. P4P strategies that align reimbursement allocation with “free market” type shifts in cognitive and procedural care using evidence-based data and positive reinforcement are more likely to produce large-scale improvements in quality and cost efficiency with respect to clinical urologic care. This paper reviews current paradigms and, using BPH procedural therapy outcomes, cost, and reimbursement data, makes the case for a fundamental change in perspective to *value-based pay for performance* as a reimbursement system with the potential to align the interests of patients, physicians, and payers and to improve global clinical outcomes while preserving free choice of clinically efficacious treatments.

## 1. INTRODUCTION

The concept of “pay for performance” applied to the practice of medicine has become a major foundation in current public and private payer reimbursement strategies for both institutional and individual physician providers. The number of pay for performance initiatives is growing nationwide [1]. The goal of these programs is ostensibly to improve quality of care and to reduce healthcare costs by linking clinical outcomes metrics with reimbursement allocation paradigms. In attempting to achieve this objective, “pay for performance” programs represent a substantial shift from traditional service-based reimbursement to a system of performance-based provider payment using financial incentives to drive improvements in the quality of care.

The current fee for service system is designed to reimburse healthcare providers based principally on the volume of services provided, without consideration for the quality of clinical outcomes. Clearly, though, the face of health care resource allocation is changing and payers are beginning to scrutinize the costs and benefits of various treatment options. Further, outcomes research and performance-based reimbursement initiatives are underway which will change the basis by which the performance of physicians, ancillary health professionals, and health care institutions are assessed by payers [2]. Although many questions exist regarding the introduction of a pay for performance (P4P) system into the field of urology, this new powerful movement cannot be ignored.

Unfortunately, the majority of currently established quality measures are designed primarily for primary care physicians and institutional providers such as hospitals. Initial performance measures that could be applied to urological patients have been released by CMS as part of its physician voluntary reporting progress (PVRP) program [3]. Examples include the use of thromboembolic and antibiotic prophylaxis in surgical patients [3]. However, these P4P strategies currently embody rudimentary *structure* and *process* (as opposed to *outcomes*) metrics which set relatively low-performance thresholds [3]. Further, current P4P quality assessment programs are generally structured using negative reinforcement strategies which penalize providers with lower reimbursement when performance standards are not met, but which often keep maximum attainable reimbursement at predefined par values [4]. Examples include “straight bonus”, “at-risk financial”, and “tiered copayment” models which may be either “competitive” (bonus payments distributed to providers based on performance stratifications) or “noncompetitive” (bonus payments distributed to all providers who meet a particular performance standard) [4].

The net result is that initial trials at P4P set a “low bar” for quality and cost efficiency and, therefore, are likely to produce only nominal improvements in clinical outcomes measures with limited global impact [3]. Moreover, rudimentary P4P metrics are unlikely to differentiate individual providers in a meaningful way on the basis of clinical outcomes. Conversely, P4P strategies that align reimbursement allocation with “free market” type shifts in cognitive and procedural care using evidence-based data and positive reinforcement are more likely to produce large-scale improvements in quality and cost efficiency with respect to clinical urologic care.

## 2. BPH EVIDENCE-BASED REVIEW OF COMMON PROCEDURAL TREATMENT OPTIONS

The management of benign prostatic hyperplasia (BPH) can serve as an excellent example of how effective P4P paradigms might be structured and implemented in urologic practice to generate meaningful improvements in clinical outcomes. BPH is one of the most common medical conditions in the aging male population in the US [5]. As pharmacologic and procedural therapies for this disease evolve, myriad studies evaluating clinical efficacy and cost effectiveness have been published. This body of research provides a unique lens through which the problem of implementing P4P principles in urology can be viewed.

Transurethral resection of prostate (TURP) is generally the gold standard treatment for BPH against which the clinical efficacy of emerging technologies is measured. In 2003, Hoffman et al. published a systematic review of randomized controlled trials to determine the clinical efficacy of TURP [6]. The pooled mean percentage improvement for urinary symptoms ranged from 63% to 77% with standard transurethral resection, while improvement for peak urinary flow ranged from 96% to 127%. In a comprehensive review of clinical outcomes and costs for BPH procedural therapy which included an analysis of the AUA BPH guideline dataset, researchers reported average TURP improvements from baseline AUA symptom score, maximum uroflowmetry rate, and quality of life scores of 66%, 117%, and 73%, respectively, 2 years after treatment [7].

Complications of TURP are well documented in the literature. Rassweiler et al. recently updated the rates of complications for TURP, including information on management and prevention based on technological evolution [8]. In that study, technological improvements such as the use of microprocessor-controlled units, the implementation of video-assisted TURP, and comprehensive training helped to reduce perioperative complications (recent versus early) including transfusion rate (0.4% versus 7.1%), TUR syndrome (0.0% versus 1.1%), clot retention (2% versus 5%), and urinary tract infection (1.7% versus 8.2%). However, a comprehensive outcomes review by Rhee et al. showed that TURP is associated with a substantial risk of complications including blood transfusion, TUR syndrome, urinary incontinence, infection, impotence, ejaculatory dysfunction, irritative voiding symptoms, stricture, hematuria, urinary retention, and the need for retreatment which effects both treatment efficacy and the cost of care [9].

Evidence-based studies that analyze total cost of care for procedural BPH therapies are also available. One sophisticated peer reviewed economic analysis estimated the total payer cost over a 2 year period from the time of treatment, including the cost of initial treatment, follow-up care, adverse events, and procedural retreatment. According to this study, the 24-month total procedural cost of TURP was $4927 [7]. With regards to physician reimbursement, the Medicare 2008 fee schedule figure is approximately $783.11 for the common hospital-based site of service (Table 1, 2008 Medicare Payment Schedule).

Laser vaporization of the prostate (PVP and HoLAP) has gained popularity due to the ability of this procedure to ablate large volumes of prostate tissue with decreased risk of complications. Additional advantages include reduced need for hospitalization, shorter catheterization times, and excellent clinical efficacy compared to standard procedural therapies and emerging minimally invasive treatments. Rajbabu et al. studied the efficiency of photoselective vaporization of the prostate (PVP) with the potassium titanyl phosphate (KTP) laser in men with prostates of >100 grams [10]. The mean maximum urinary flow rate (standard deviation) improved from 8.0 (3.1) to 18.2 (8.1), 18.5 (9.2), 17.9 (7.8), and 19.3 (9.8) mL/s at 3, 6, 12, and 24 months, respectively. The IPSS and quality of life (QoL) scores showed similar improvements. There were no major complications, and no patient developed transurethral resection syndrome or required a blood transfusion [10]. Tan et al. have also published on the clinical efficacy of this technique [11].

Other researchers have performed formal meta-analysis of KTP laser vaporization demonstrating improvements in symptom score, maximal flow rate, and QoL scores of 76%, 221%, and 83% at 24 months, respectively [7]. Further, when compared to ILC, TUNA, TUMT, and TURP, laser vaporization displayed very low adverse event rates including the risks of incontinence, UTI, impotence, ejaculatory dysfunction, irritative voiding, bladder neck stenosis/stricture, urinary retention, hematuria, and reoperation [7].

These investigators estimated the 24-month total procedural cost, including expenditures for initial treatment, follow-up care, adverse events, and procedural retreatment to be $3589 [7]. The 2008 Medicare fee schedule reimbursement for laser vaporization differs significantly based on the site of service with approximate physician fee schedule reimbursement of $648.11 and $2498.50 for procedures performed in the hospital and office settings, respectively (Table 1, 2008 Medicare payment schedule).

Interstitial laser coagulation (ILC) of the prostate can be performed in the hospital inpatient, hospital outpatient, ambulatory surgery center, or office settings under local, regional, or general anesthesia. In 1998, Williams reported the introduction of a volume-based treatment formula with 12-month follow-up [12]. In this study, treatment outcomes were evaluated at 3-, 6-, and 12-month intervals using the American Urological Association (AUA/IPSS) BPH symptom score, maximal urinary flow, prostate size, and postvoid residual urine volume. The AUA symptom score decreased from 23.2 (range 17–28) prior to treatment to 9.4 (range 4–14) at 3 months, 6.6 (range 5–12) at 6 months, and 7.2 (range 4–11) at 12 months. The maximal flow rate improved from 8.4 (range 5–10) milliliters per second before treatment to 14.1 (range 10–20) mL/s at 3 months, 14.8 (range 10–18) mL/s at 6 months, and 16.8 (range 12–25) mL/s at 12 months after treatment. In addition, Williams reported no significant postprocedure complications [12]. Other investigators, in a more recent comprehensive review and meta-analysis of ILC published studies, demonstrated average improvements from baseline of 62%, 89%, and 55% for AUA symptom score, maximum uroflowmetry rate, and the quality of life score, respectively, at 24 months posttreatment [7]. Unfortunately, ILC has demonstrated a relatively high risk of reoperation with one study reporting 16% of men requiring TURP within 2 years of initial treatment of 16% [13].

In comparison, another peer-reviewed report using formal meta-analysis techniques demonstrated relatively high adverse event rates and a weighted average probability of retreatment of 10% along with a 24-month total procedural cost (including cost of initial treatment, follow-up care, adverse events, and procedural retreatment) of $4754 [7]. As with laser vaporization, the 2008 Medicare physician reimbursement depends on the site of service in which the procedure is performed. In that regard, the approximate physician reimbursement for ILC is $606.77, and $2457.86 for the hospital and office settings, respectively (Table 1, 2008 Medicare payment schedule).

Transurethral Radiofrequency needle ablation (TUNA) of prostate is generally performed in the outpatient setting under local or regional anesthesia. Bouza et al. performed a systematic review and meta-analysis of TUNA in symptomatic BPH [14]. Although evidence was limited by methodological issues, the analysis indicated that while TUNA significantly improved BPH parameters with respect to baseline, these improvements did not match those achieved with TURP. In addition, the clinical efficacy declined in the long term with a retreatment rate significantly higher than that of TURP [14].

In a review and meta-analysis of minimally invasive BPH procedural therapies, TUNA demonstrated improvements from baseline AUA symptom score, maximum uroflowmetry rate, and quality of life scores of 52%, 35%, and 68%, respectively, at 6 months after treatment [7]. These rates declined at 24 months follow up to 44% improvement in symptom score and 28% improvement in uroflowmetry rates. However, the improvement of quality of life score appeared to be maintained at 61% [7]. This report also demonstrated the probabilities of adverse events for the procedure to be significant, including a 31% rate of dysuria/irritative voiding symptoms, 20% risk of urinary retention, and 17% chance of urinary tract infection. The weighted average reoperation rate reported in this study was 23%, the highest of all treatment modalities reviewed [7].

The 24-month total procedural cost including expenditures for initial treatment, follow-up care, adverse events, and procedural retreatment has been estimated at $6179 [7]. An analysis of 2008 Medicare physician reimbursement for TUNA shows approximate values of $582.43 and 2891.72 for the facility and nonfacility settings, respectively. In practice, however, the procedure is generally performed in the office site of service by most physicians (Table 1, 2008 Medicare payment schedule).

Transurethral microwave therapy (TUMT) of the prostate also affords the advantage of being performed in the office setting using minor sedation and local anesthesia. In evaluating clinical measures of efficacy, Hoffman et al. performed a review of randomized controlled trials to evaluate urinary symptom improvement, urinary function, prostate volume, mortality, morbidity, and retreatment rates [15]. In that evaluation, the pooled mean urinary symptom scores decreased by 65%, while the pooled mean peak urinary flow rates increased by 70%. Compared to TURP, TUMT was associated with decreased risk of retrograde ejaculation, the formation of clinically significant strictures and hematuria, the need for blood transfusions, and the development of transurethral resection syndrome. However, the analysis showed an increased risk for posttreatment dysuria and urinary retention as well as the need for retreatment related to deterioration in BPH symptoms [15].

An analysis of reported outcomes data from the comprehensive AUA BPH guidelines demonstrated 39%–46% symptom score improvement at 24 months [7]. Maximal uroflowmetry rate and quality of life score improvements ranged from 28%–45% and 24%–52% at 24 months, respectively [7]. The weighted average probability of an adverse event varied based on the equipment used, with a 74% rate of dysuria/irritative voiding for the Prostatron Version 2.5 versus 28% when using the Prostatron Version 2.0. The rates of reoperation again varied depending on the system used, ranging from 10% to 16% [7]. Other commonly reported adverse events included incontinence (range 2–6%), UTI (9%), impotence (range 1–3%), stricture (range 1–3%), and retention (range 6–23%) [7].

In that study, the expected cost per patient at 24 months, including expenditures for initial treatment, follow-up care, adverse events, and procedural retreatment was $5461 when using Prostatron 2.0, $5488 with Prostatron 2.5 and $5699 with the Targis system [7].

A review of 2008 Medicare fee schedule physician reimbursement for TUMT indicates an approximate value of $2901.75 in the office site of service and $536.34 for a hospital-based procedure (Table 1, 2008 Medicare payment schedule). However, virtually, all TUMT procedures are currently performed in the office outpatient setting.

Laser enucleation of the prostate (HoLEP) is generally performed in the hospital outpatient or inpatient setting and provides a relatively minimally invasive means of removing a broad range of obstructing prostate adenomas. Studies of clinical efficacy have documented profound improvements in outcomes metrics including symptom score, quality of life score, and uroflowmetry rates that compare favorably to the gold standard TURP procedure [16, 17]. In addition, HoLEP tends to produce relatively low-adverse event rates including objective assessments for the risks of transfusion, impotence, irritative voiding, retention, incontinence, stricture, and UTI compared to other BPH ablation procedures [16–19]. However, HoLEP carries a relatively high risk of bladder mucosal injury and a steep learning curve to achieve technical competence relative to other transurethral therapies. While meta-analytic cost data comparing HoLEP to other procedural BPH therapies is not generally available, the approximate 2008 Medicare fee schedule physician reimbursement for this procedure is $951.99 utilizing a hospital site of service (Table 1, 2008 Medicare payment schedule).

## 3. STRUCTURING PAY FOR PERFORMANCE IN UROLOGY: THE BPH EXAMPLE

Armed with a thorough understanding of clinical outcomes, cost, and reimbursement data for commonly performed BPH procedural options, it is now possible to examine opportunities for developing P4P programs in urology using this disease entity as an example. To be sure, few would dispute the conceptual value of transforming the current system of medical service reimbursement with a more rationale, evidence-based approach. The challenge in proposing a P4P scheme for BPH procedural therapy is to understand the method by which physician reimbursement for professional services is calculated and the role of this system in influencing clinical outcomes. Then, a proposal can be developed which outlines potential ways that physician payment allocation might be restructured to drive aggregate physician decision making toward the selection of procedural services that result in improved outcomes while also preserving free choice from a market basket of therapeutic alternatives with heterogeneous clinical and economic performance characteristics.

The first step in proposing an effective pay for performance initiative for BPH procedural therapy is to internalize the complexity of the calculation used to formulate Medicare reimbursement for specific treatment options. At its core, the resource-based relative value scale system for physician payments (RBRVS) was developed from the principle that the “relative value” of physician services is comprised of 3 essential components: (1) the physician work expended performing a medical service, (2) the practice expense attributable to providing that service, and (3) the component of malpractice expense that can be allocated to the distinct procedural or cognitive care [20]. Total Medicare health care expenditures are limited based on the sustainable growth rate (SGR) method which is, in turn, linked to changes in the gross domestic product (GDP) [20]. Critical flaws of the current system of determining physician payments for medical services include problems with the calculation of actual work input values for specific services, the inconsistent allocation of practice expenses for hospital, ambulatory surgery center and office-based care, and the coupling of health expenditures with GDP which may not reflect inflationary pressures specific to the practice of medicine. However, most important to the discussion of P4P implementation in urology is the total absence of clinical and economic outcomes measures or performance standards in the RBRVS calculation.

A review of the previous analysis of outcomes, cost, and physician payment data for BPH procedural treatment in the context of the RBRVS methodology provides an unambiguous view of the way that reimbursement strategies can affect clinical outcomes. From this review, two major points are evident. First, physician reimbursement for BPH procedural therapy demonstrates marked variation based primarily on differences in key inputs to the RBRVS calculation which are impacted by the technologies employed and the site of service utilized in performing these procedures. Second, even allowing for adjustments based on procedural overhead cost, there is clear misalignment between evidence-based clinical outcomes measures and current Medicare fee schedule physician reimbursement for commonly performed BPH procedures.

This misalignment creates an environment in which choices between objective outcomes measures and physician reimbursement are in competition during the process of BPH procedural treatment selection. In this way, the current reimbursement paradigm for BPH procedural therapy actually works to select for less optimal clinical outcomes as physicians are forced into an awkward position, where rational decisions cannot be made on the basis of clinical outcomes and reimbursement at the same time. To be sure, other factors should also play a substantial role in determining the appropriate BPH procedural treatment for a given patient. Proper treatment selection should reflect sound joint decision making and consumer-centered care that reflects both the physician's use of evidence-based information and the incorporation of patient specific factors including the relative importance of adverse event rates, comorbidities, location of service, objective clinical improvement, and cost. However, by placing reimbursement and outcomes in direct opposition, the current volume-based methodology for allocating physician reimbursement for BPH therapies creates a sizeable barrier to the development of a workable pay for performance program.

Addressing the challenge of proposing a viable P4P paradigm for BPH procedural treatment or any other medical service requires several important steps. First, policymakers must fix the RBRVS/SGR methodology to resolve recurring problems with the econometric formula including, but not limited to, inaccuracies in the estimation of the practice expense component for procedural reimbursement and the flawed link between physician payment calculations and variation in the GDP [20]. Second, the RBRVS formula should be modified to embody clinical outcomes performance metrics using, for example, additional factors that reflect the relative difference between individual procedures and services based on measurable evidence based data. Finally, key reimbursement decision makers should change the frame of reference from a punitive system of individual physician performance assessment to a “supply side” approach which utilizes a realignment of physician payments with outcomes to influence *aggregate physician-patient joint decision making.*

The potential benefits to structuring an effective pay for performance paradigm for BPH procedural therapy are enumerable. Effective treatment selection can have an enormous impact on downstream outcomes indicators including the degree of clinical improvement and the risk of adverse events which, in turn, largely determine the real cost of care [7]. Further, pay for performance methodologies that use physician reimbursement as an incentive to *value-based* (as opposed to volume-based) decision making are more likely to result in sustainable quality improvement [20]. Properly structured P4P programs can then form the foundation upon which effective reimbursement strategy is built.

Conversely, punitive P4P programs that use rudimentary *structure* and *process* metrics are unlikely to produce lasting quality or cost improvements as these methodologies do little to promote competition based on the overall value of medical services provided [20]. Moreover, these crude measures create performance standard thresholds that are relatively easy to achieve and that promote the tendency to shape clinical decision making simply to satisfy a particular quality metric [3]. The net result of P4P programs based on individual physician performance assessment is a tendency toward interval scale rankings that are unable to accurately differentiate providers based on quality of care and which do not address the misalignment of reimbursement and clinical outcomes present in the current payment paradigm for BPH procedures.

Hypothetically, physician reimbursement for BPH procedures can be structured in at least 3 basic ways. At one extreme, physician payments can be allocated without incorporating clinical outcomes measures in the calculation. This case example results in the current system, where the choice from among competing alternatives is based on a number of interrelated factors. In this scenario, while procedures with superior efficacy will sometimes be chosen, clinical outcomes in the aggregate will never be optimized as competing incentives allow less efficacious treatments to be selected disproportionately. At the other extreme, physician payments can be allocated equally among the various treatment alternatives. This case would result in an environment, where there is no direct economic incentive to choose any one BPH treatment. In this scenario, treatment decision making may result in the selection of procedures with healthier outcomes characteristics but the lack of economic incentives would continue to result in a subset of providers utilizing less efficacious alternatives for other reasons including differences in the learning curve to master particular techniques and the capacity of some technologies to be more amenable to volume-based practice.

In the end, *value-based P4P* is the only practicable way to appropriately structure BPH procedural treatment selection to improve clinical outcomes by aligning the interests of physicians, patients, and payers with rational reimbursement policy. In this case, physician payments would be weighted and allocated based on a validated analysis of evidence-based outcomes data. BPH procedures that exhibit better outcomes performance characteristics would be allocated greater physician reimbursement and competition between reimbursement and outcomes would be curbed. Moreover, *value-based P4P* that utilizes clinical outcomes as the core performance measure, as opposed to punitive structure and process metrics, would move away from individual physician performance assessment with its inherent limitations to a system that maintains free choice from among the available treatment alternatives while at the same time providing market-based positive reinforcement to *globally influence aggregate physician decision making* toward procedures with superior outcomes characteristics (in much the same way, for instance, as the federal reserve shapes financial decisions through manipulations of key interest rates and the money supply). In this way, the frame of reference and focus appropriately shifts *from* the individual provider *to* the therapies provided and allows physicians and patients to make rational, evidence-based treatment decisions.

Certainly, the rate limiting step in the development of *value-based P4P* for BPH procedural treatment or any other medical condition is the availability of clinically meaningful outcomes criteria which can be populated with uniform evidence-based data [3]. However, previous research has indeed shown that high quality meta-analysis of AUA guideline and other peer-reviewed data can be performed and meshed with Medicare cost figures to compare BPH procedures in an unbiased fashion [7]. Further, *value-based P4P* can integrate more sophisticated metrics including *“total cost of care”* which by incorporating factors such as the costs of initial therapy, follow-up care, management of adverse events, and retreatment can act as a valuable measure of clinical efficacy [7].

In conclusion, doctors and other providers should be rightly cautious about current P4P initiatives that use rudimentary metrics and punitive reimbursement policy and which fail to differentiate participants or improve aggregate clinical outcomes. However, urologists, patients, and payers should embrace the concept of *value-based pay for performance* as a reimbursement system with the potential to align the interests of these distinct constituencies and to improve global clinical outcomes while preserving free choice of clinically efficacious treatments.

## Figures and Tables

**Table 1:** *2008 Medicare physician payment data for BPH procedural therapies* (figures are derived using the CMS listing for the 2008 fee schedule, the 2008 conversion factor, and the RBRVS formula).

CPT code/procedure	Nonfacility Medicare payment schedule 2008	Facility Medicare payment schedule 2008
52601 (TURP complete)	N/A	$783.11
52647 (Indigo laser)	$2457.86	$606.77
52648 (contact laser vaporization of prostate)	$2498.50	$648.11
52649 (laser Enucleation of the Prostate)	N/A	$951.99
55821 (prostatectomy, suprapubic simple)	N/A	$829.45
53850 (microwave thermotherapy)	$2901.75	$536.34
53852 (radiofrequency ablation)	$2891.72	$582.43

## References

[1] Casalino L, Gillies RR, Shortell SM (2003). External incentives, information technology, and organized processes to improve health care quality for patients with chronic diseases. *Journal of the American Medical Association*.

[2] Brook RH, McGlynn EA, Shekelle PG (2000). Defining and measuring quality of care: a perspective from US researchers. *International Journal for Quality in Health Care*.

[3] Gonzalez CM, Penson D, Kosiak B, Dupree J, Clemens JQ (2007). Pay for performance: rationale and potential implications for urology. *The Journal of Urology*.

[4] Miller DC, Wei JT, Montie JE, Hollenbeck BK (2006). Quality of care and performance-based reimbursement: the contemporary landscape and implications for urologists. *Urology*.

[5] Taub DA, Wei JT (2006). The economics of benign prostatic hyperplasia and lower urinary tract symptoms in the United States. *Current Urology Reports*.

[6] Hoffman RM, MacDonald R, Slaton JW, Wilt TJ (2003). Laser prostatectomy versus transurethral resection for treating benign prostatic obstruction: a systematic review. *The Journal of Urology*.

[7] Stovsky MD, Griffiths RI, Duff SB (2006). A clinical outcomes and cost analysis comparing photoselective vaporization of the prostate to alternative minimally invasive therapies and transurethral prostate resection for the treatment of benign prostatic hyperplasia. *The Journal of Urology*.

[8] Rassweiler J, Teber D, Kuntz R, Hofmann R (2006). Complications of transurethral resection of the prostate (TURP)—incidence, management, and prevention. *European Urology*.

[9] Stovsky MD, Rhee K, Hartke D (2007). Medical therapy versus surgery and minimally invasive surgical therapies for lower urinary tract symptoms and benign prostatic hyperplasia: what makes better economic sense?. *Current Urology Reports*.

[10] Rajbabu K, Chandrasekara SK, Barber NJ, Walsh K, Muir GH (2007). Photoselective vaporization of the prostate with the potassium-titanyl-phosphate laser in men with prostates of >100 mL. *BJU International*.

[11] Tan AHH, Gilling PJ, Kennett KM, Fletcher H, Fraundorfer MR (2003). Long-term results of high-power holmium laser vaporization (ablation) of the prostate. *BJU International*.

[12] Williams JC (1998). Interstitial laser coagulation of the prostate: introduction of a volume-based treatment formula with 12-month follow-up. *World Journal of Urology*.

[13] Kursh ED, Concepcion R, Chan S, Hudson P, Ratner M, Eyre R (2003). Interstitial laser coagulation versus transurethral prostate resection for treating benign prostatic obstruction: a randomized trial with 2-year follow-up. *Urology*.

[14] Bouza C, López T, Magro A, Navalpotro L, Amate JM (2006). Systematic review and meta-analysis of Transurethral Needle Ablation in symptomatic benign prostatic hyperplasia. *BMC Urology*.

[15] Hoffman RM, Monga M, Elliot SP, MacDonald R, Wilt TJ (2007). Microwave thermotherapy for benign prostatic hyperplasia. *Cochrane Database of Systematic Reviews*.

[16] Gupta N, Sivaramakrishna, Kumar R, Dogra PN, Seth A (2006). Comparison of standard transurethral resection, transurethral vapour resection and holmium laser enucleation of the prostate for managing benign prostatic hyperplasia of >40 grams. *BJU International*.

[17] Wilson LC, Gilling PJ, Williams A (2006). A randomised trial comparing holmium laser enucleation versus transurethral resection in the treatment of prostates larger than 40 grams: results at 2 years. *European Urology*.

[18] Elzayat EA, Habib EI, Elhilali MM (2005). Holmium laser enucleation of the prostate: a size-independent new “gold standard”. *Urology*.

[19] Montorsi F, Naspro R, Salonia A (2004). Holmium laser enucleation versus transurethral resection of the prostate: results from a 2-center, prospective, randomized trial in patients with obstructive benign prostatic hyperplasia. *The Journal of Urology*.

[20] O'Shea JS (2008). Medicare physician payment reform: changing incentives to maintain access to quality surgical services. *Journal of the American College of Surgeons*.

